# Editorial: Emerging Swine Viruses

**DOI:** 10.3389/fvets.2020.00132

**Published:** 2020-03-24

**Authors:** Carlos Juan Perfumo, Ariel Pereda, Anan Jongkaewwattana, Zhenhai Chen, Daniel Roberto Perez, Jingyun Ma

**Affiliations:** ^1^Faculty of Veterinary Sciences, La Plata National University, La Plata, Argentina; ^2^Instituto Nacional de Tecnología Agropecuaria Laboratorio de Virologia de Aves y Cerdos, Buenos Aires, Argentina; ^3^National Center for Genetic Engineering and Biotechnology (BIOTEC), Pathum Thani, Thailand; ^4^College of Veterinary Medicine, Yangzhou University, Yangzhou, China; ^5^Department of Population Health University of Georgia, Athens, GA, United States; ^6^College of Animal Science, South China Agricultural University, Guangzhou, China

**Keywords:** editorial, swine, emerging, viruses, diseases

Over the last 30 years, diseases caused by emerging swine viruses (ESV) have acquired great relevance, more than in other species. Diseases caused by porcine reproductive and respiratory syndrome virus (PRRSv), high pathogenicity porcine epidemic diarrhea virus (PEDv), porcine circovirus type 2 (PCV-2), and influenza virus H1N1pdm09 had great economic impact. Others, however, such as porcine enteroviruses, porcine toroviruses (PToV), porcine sapelovirus (PSV), porcine bocavirus (PBoV), porcine kobuvirus (PKBV), and porcine Torque teno sus virus (TTSuV) are mostly subclinical in swine herds. Furthermore, novel emerging viruses, such as SENECA virus, atypical porcine pestivirus (APPV), PCV-3, SADS-CoV, influenza D, and others with regional or worldwide distribution constitute a new challenge for researchers and practicing veterinarians.

Emerging viruses should be considered to occur when there are changes in the relationship between the agent, the host and the enviroment. The response to how and why the ESV have emerged can be explained through several factors.

First, interspecies transmission means the presence of a potentially pathogenic agent into a new host, such as between aquatic migratory birds and human beings for influenza A. Bats are the source of Nipah virus, and swine acute diarrhea syndrome (SADS coronavirus). Both have limited distribution to Asia or TGE and PED. Currently PCV-3 have been found with high homology with bats PCV-1.

Secondly, changes in the virulence (mutation, reassortant, recombination) of the agents in the same host, particularly the RNA and single strand DNA viruses that have a high mutation rate (10–4/10–5 nucleotides per replication cycle), that facilitate its adaptation to the innate immune response. The absence of enzymes (transcriptase) in infected cells that correct errors in reading RNA synthesis and segmented RNA chains favor reassortant. A population of RNA viruses does not consist of a single genotype, but a “set or cloud” of related viruses that interact with each other called “quasispecies”. Relevant examples are HP PRRSv, influenza A H1N1pdm09 and PEDv.

Next, the viruses have been present for a long period of time as subclinical infections and have been discovered with the development of metagenomic techniques [Next Generation Sequencing NGS, Lawrence Livermore Microbial Detection Array (LLMDA or virochip)], or exogenous factors as most emerging viruses do not grow in traditional culture media. Viruses such PCV-3, SADS-CoV, and LINDA virus have been characterized by the aforementioned techniques.

Now we come to change in the production system. The presence of farms of good health, large size and homogeneous genetics favors the fitness, and the ability of a particular population of viruses to multiply and spread in a specific environment.

Our fifth point is the recognition and sensitization of practicing veterinaries of “abnormal” cases or syndromes through the routine postmortem monitoring of pigs that die “unexceptionally” on the farm, as well as the syndromes surveillance at the slaughterhouse.

Finally, Specialized diagnostic laboratories that offer new and accessible diagnostic tools for the diagnoses of known and unknown emerging viruses.

This Research Topics comprises ten review articles are related by: Next Generation Sequencing (NGS) plus *in situ* Hybridization (ISH); porcine hemagglutinating encephalomyelitis coronavirus (PHE-CoV); porcine circovirus type 3 (PCV-3); classical swine fever virus (CSF); porcine torovirus (PToV); porcine respiratory reproductive syndrome virus (PRRSV); human influenza A; Nipah virus in humans and pigs swine; atypical porcine pestivirus (APPV); porcine epidemic diarrhea coronavirus (PEDV); and porcine delta coronavirus (PDCoV) pathogenesis. These subjects provide a discussion on the broad field of emerging swine viruses infections and its control.

Three original research articles about phylogeny and genome composition of novel Seneca virus (SVA) isolated in China; estimation of time to porcine epidemic diarrhea coronavirus (PED-CoV) removal in Ontario herds and a longitudinal serological and RT-PCR fecal studies of hepatitis E virus (HEV) were published.

## Review Articles

Resende et al. provide comprehensive information about the results of the combination of NGS-ISH for the diagnosis of known and unknown emerging pathogens in tissues by NGS, and its relationship with specific lesions where it is visualized in active infection through detection of mRNA by ISH. Results on the application of PCV-2, PPV-2, Seneca virus, and *Mycoplasma hyorhinis* are comments.

Mora-Díaz et al. review the disease produced by porcine hemagglutinating encephalomyelitis coronavirus (PHE-CoV), a neurotropic virus affecting piglets <4 weeks old. Subjects such as: characteristic of the virus, history of the emergence of PHE-CoV, global distribution, clinical signs, pathogenesis, lesions, and diagnosis are discussed. As the infection is endemic in most swine herds, and no current vaccines are available, early exposure to old or young sows to induced maternal immunity is the only way to prevent the disease.

Klaumann et al. discuss the current knowledge on a new circovirus named porcine circovirus type 3 (PCV-3). Originally this was identified by metagenomic analyses from an outbreak of PDNS in sows associated with reproductive failure, myocarditis, and multisystemic inflammation. Thereafter it was found associated with respiratory, digestive and nervous signs in healthy pigs and wild boars. Retrospective studies detected PCV-3 as early as the 1990s. Coinfection with several virus and bacteria were reported. The authors emphasize the need of studies related to pathogenesis, the role of coinfections and their association or not with certain clinically pathological entities.

Zhou reviews classical swine fever (CSF) in China. The author discusses the epidemiology, and the geographical distribution of genotypes where 2.1, 2.1b, and 2.1c are currently dominant in China. The first one persists in an immune population by natural infection due to the high mutation rate of the enveloped glycoprotein E2. For eradication of CSF it is necessary to distinguish between the naturally infected and vaccinated animals by live attenuated marked vaccine. An experimental E2 subunit vaccine was developed in China. Besides preventive vaccination, we need culling strategies, skilled veterinarians, up-to-date diagnostic and monitoring technology, and biosecurity.

Hu et al. review the progress in the knowledge on porcine torovirus (PToV) a single-stranded RNA enteric virus found in piglets with diarrhea in North America, Africa, Asia, and Europe. The authors describe the virus morphology, the genomic structure and genotypes division, although chimeric strains with genes from porcine and bovine ToV has been identified as well as recombination with enterovirus. For epidemiological studies an indirect ELISA based in recombinant N protein expressed in baculovirus is available. Other methods included RT-PCR; qRT-PCR; and nested PCR. Prevalence of PToV is quite variable according with the country.

Montaner-Tarbes et al. analyze numerous gaps in PRSSv knowledge. Related with the biology, the scarce whole genome sequencing from different geographical origins hinder understanding the virus evolution/mutation. The function and the complex interaction of viral non-structural proteins with the target cells are reviewed. The mechanisms of the virus to avoid the innate and acquired immune response through recognition and antibody neutralization are reviewed. The new known mechanisms of dissemination mediated by cell to cell connected nanotubules and extracellular vesicles are thoroughly discussed. Later on, the development of exosomes, as a novel vaccine is analyzed.

Rajao et al. review the role of pigs in the interspecies transmission and how their susceptibility to different viruses can affect the overall epidemiology of swine influenza. The factors that have been implicated in the interspecies transmission of influenza such as receptor-binding specificity/affinity, balance between HA and NA content, host temperature and host-specific immune factors are analyzed. Surveillance of IAV in swine has shown that human viruses are transmitted to pigs more frequently than from pigs to humans. The result is the establishment of several human-origin virus lineages, antigenic diversity and failure of current swine vaccines.

McLean and Graham provide an update about Nipah virus (NiV), an RNA paramyxovirus that causes a severe neurological disease in humans. In suckling pigs, NiV infection causes high mortality and in older pigs, respiratory and neurological signs. The natural host of NiV is a fruit bat of the genus *Pteropus*, and pigs act as an “amplifying host”. The disease has been found in Asia in people with close contact to pigs. Recombinant NiV mutant, attenuated and subunit vaccine using several viral vectors have been studied. Currently, neither a human nor a pig vaccine has been licensed.

Gatto et al. review the information around a new RNA Pestivirus named atypical porcine pestivirus (APPV), detected in pigs with congenital tremors (CT) type AII, and splayed legs in offspring from sows by NGS technology. Viral genomes were detected in semen, preputial swabs and fluids highlighting the importance of AI in APPV epidemiology. Horizontal transmission can be made by surviving CT in healthy new-born boars, piglets and adult pigs. The virus exhibits high genetic diversity. Recently, APPV was detected in wild boars. Until the development of a vaccine, the authors recommend feedback on reproductive management in sows with CT cases.

Koonpaew et al. review the emergence of highly pathogenic porcine epidemic diarrhea (PED) and porcine delta coronavirus (PDCoV) as agents of watery diarrhea in suckling piglets. The authors describe aspects related to the biology pathogenesis and the host innate immune response of gastrointestinal tracts against those enteric coronaviruses. The agents evade recognition by host pattern recognition receptors (PRRs), present in resident antigen present cells (APCs) and located in gut associated lymphoid tissue (GALT), through the inhibition or blocking of interferon (IFN) induction and the signaling cascade, respectively. This knowledge will profit the development of immune modulators as well as effective vaccines.

## Original Research

Sun et al. analyze the phylogeny and genome compositions of 17 novel Seneca virus (SVA) isolated in China in 2017 and compare them with the genomic sequences deposited in the GenBank. SVA is a single stranded positive-sense RNA virus associated with porcine idiopathic vesicular disease (PIVD), and sudden neonatal dead reported in six countries in Asia and America. The isolated strain clustered into three distinct groups: A, B, and C, not related with the previously SVA identified in China and different from SVA identified in other countries. More effort should be directed to SVA monitoring, rapid and specific diagnosis and vaccination strategies.

Perri et al. studied the estimation of time to eliminate porcine epidemic diarrhea coronavirus (PED-CoV) in Ontario herds based in large-scale disease control program database (DCP). The analysis takes into consideration the time between the initial infection, and the confirmation of PED-CoV freedom at the minimum level of 10%. The median time to elimination varied from 23 weeks in nursery herds, to 43 weeks in farrow-to-feeder herds. Farrow-to-wean herds had the highest hazard of PED-CoV elimination. Type of herds, season and year of original diagnosis were associated with the time of negativity and reflect the complexity of the infection control practices.

Krog et al. work to determine the dynamics of infection of hepatitis E virus (HEV) by carrying out a longitudinal serological and RT-PCR fecal studies. Sows and their progeny from 2 weeks to slaughter were sampled. Antibodies were only detected in offspring born from sows with high levels of maternal antibodies (MAbs) and a few of them became shedders. All pigs seroconverted at 13–17-week-olds. By PCR 65.5% of pigs were positive at least one time during the weeks 13, 15, and 17. In 3 out of 10 slaughter pigs, HEV was detected in feces and organs. As MAbs reduced the shedder of HEV, sow's vaccination might be an option.

As a summary, this Research Topic provides a comprehensive review about the results of the combination of NGS-ISH for the diagnosis of known and unknown emerging pathogens. It's also an important discussion of potentially emerging viruses such as PCV-3, torovirus, atypical pestivirus, reemerging viruses such as PHECoV and transboundary viruses such as classical swine fever. The mechanisms used by PRRS to circumvent the host's innate immune and vaccine immune response are updated along with the development of vaccines to exosomes. The immune response against enteric coronaviruses such as PED and PDCoV and innovative vaccines for both viruses are analyzed. The role of pigs as an amplifier of the Nipah virus is reviewed, as well as the importance of vaccination to pigs for the prevention of this infection in man. The repeated transmission of human seasonal viruses to pigs has resulted in the establishment of several human-origin virus lineages globally and the failure of the current pig vaccines. A research study indicated that isolated strain Seneca virus from vesicular fluid of sows, clustered into three distinct groups, A, B, and C not related with the previously SVA identified in China, and highlight that different genotypes of SVA co-exist and spread. A study of the estimation of time to porcine epidemic diarrhea coronavirus (PED-CoV) elimination in Ontario Type of herds was carried out. The year and season of original diagnoses are associated with the time of negativity and reflect the complexity of the infection control practices. Finally, a study shows that hepatitis E virus infection is widespread in the herd, and pigs spread virus during the final stages of life, and there's a strong chance to find infected pigs at slaughter. As maternal antibodies reduce, the shedder of virus vaccinating sows might be an option.

## Author Contributions

All authors listed have made a substantial, direct and intellectual contribution to the work, and approved it for publication.

### Conflict of Interest

The authors declare that the research was conducted in the absence of any commercial or financial relationships that could be construed as a potential conflict of interest.

